# Predictors of never having a mammogram among Chinese, Vietnamese, and Korean immigrant women in the U.S.

**DOI:** 10.1371/journal.pone.0224505

**Published:** 2019-11-06

**Authors:** En-Jung Shon, Aloen Louise Townsend

**Affiliations:** 1 Department of Family Science and Social Work, Miami University, Oxford, OH, United States of America; 2 Jack, Joseph and Morton Mandel School of Applied Social Sciences, Case Western Reserve University, Cleveland, OH, United States of America; University of Chicago, UNITED STATES

## Abstract

**Background:**

Breast cancer is the most common cancer among Asian women in the U.S. The first objective was to investigate predictors (including ethnicity) of never having a mammogram in middle-aged and older Chinese, Vietnamese, and Korean immigrant women (main effects). The second objective was to explore whether relationships between predictors and never having a mammogram varied across the three groups (moderation effects of ethnicity).

**Methods:**

Merged (2005-2007-2009-2011) California Health Interview Survey data were utilized. Unweighted sample was 3,710 Asian women ages 40 years and older (Chinese = 1,389; Vietnamese = 1,094; Korean = 1,227). Replicate weighted total sample size was 1,710,233 (Chinese = 940,000; Vietnamese = 410,000; Korean = 360,000). Replicate-weighted multivariate logistic regression was applied. Interaction effects (moderator role of ethnicity) were also examined, using multivariate logistic regression, for the second objective.

**Results:**

For the first objective, odds of never having a mammogram were higher for women who were Korean (Ref = Vietnamese), unmarried, or a non-U.S. citizen. Odds were lower in women ages 50–59 or 60–69 (Ref = 70–85). Regarding the second objective, only for Chinese women, odds of never having a mammogram were lower as the number of physician visits got higher.

**Conclusion:**

Culturally-sensitive outreach and services should be developed to target higher-risk groups. Patient-centered healthcare strategies tailored for the three groups could be effective. For Chinese women, in particular, regular information sessions or education programs could be provided for enhancing their physician visits.

## Introduction

Breast cancer is the most common cancer among Asian women in the U.S. [1, 2] From 2005 to 2014, Asian American women’s breast cancer incidence rate increased more steeply than other racial or ethnic groups [2]. Asian American women also experienced the lowest decrease in death rate per year from breast cancer from 2005 to 2014 (0.9%), compared to 2.6% in American Indian/Alaska Native, 1.8% in non-Hispanic White, 1.5% in non-Hispanic Black, and 1.4% in Hispanic women [2]. According to the National Cancer Institute (2017), the decline in breast cancer death rate began a decade later among Asian American women than other racial and ethnic groups. These statistics highlight the greater vulnerability of Asian American women to breast cancer incidence or death [3].

Asian immigrant women (i.e., foreign born) have a consistently advanced breast cancer stage at diagnosis and, consequently, a lower survival rate than their U.S.-born counterparts [4]. California Cancer Registry data (2010) indicated that Asian immigrant women had a 2–11% lower rate of 5 year breast cancer survival compared to U.S.-born Asian women [4]. Physicians highlight that having a mammogram is essential for early detection of breast cancer, in order to reduce the likelihood of diagnosis at an advanced stage that can cause a higher death rate [2]. However, among all ethnoracial groups, Asian immigrant women (40 years and older) in the U.S. obtain mammography at the lowest rate (either in their lifetime or within 1–2 years) [5]. Asian immigrant women who have never had a mammogram during their lifetimes could be in a more vulnerable situation as they are more exposed to undiagnosed breast cancer or advanced stage of breast cancer at diagnosis.

Prior studies including diverse groups (Chinese, Vietnamese, Korean, or other, who were mostly immigrants) or a single group of Asian American women (Chinese only, Vietnamese only, or Korean only, mostly immigrants) found that those who were married [6–9], more educated [7, 8, 10], employed [11, 12], had higher annual household income [7, 13], or had a larger number of children [8] had higher odds (or higher percentage) of ever having a mammogram than their counterparts. Women who had U.S. citizenship [14], lived in the U.S. for a longer time [6, 9, 11, 12, 15–18], or perceived that they speak English well or very well [10, 11, 15, 19] also had higher odds (or higher percentage) of ever having a mammogram than their counterparts. Accessibility of healthcare resources was significantly associated with lifetime mammography among Asian immigrant women. For example, those who had health insurance [6, 12, 13, 15–17, 20], mammogram coverage [21], a usual source of healthcare [10], primary physicians to visit [15, 18, 20], regular check-ups for health [9, 22], physicians’ recommendation to have mammography [6, 7, 11, 17], or no communication problem with physicians [15] had higher odds (or higher percentage) of ever having a mammogram than their counterparts. Notably, studies targeting diverse Asian women found that Korean women had lower odds of ever having a mammogram than Filipina women [16] or higher percentage of never having a mammogram than Chinese, Vietnamese, or other Asian women [23–25].

Although prior studies targeting diverse Asian immigrant women found disparities in the odds (or prevalence) of ever/never having a mammogram, few examined what specific factors account for the ethnic variance across Asian immigrant subgroups in never having a mammogram. To prevent overlooking the specific predictors of lifetime mammography of particular Asian immigrant groups [26, 27], it is essential to consider ethnicity as both a main effect and a moderator of the relationships between predictors and never having a mammogram. In particular, as ethnicity is often linked to a combination of societal factors, environmental situations, and cultural backgrounds of individuals [28], understanding the moderating role of ethnicity will be critical.

### Theoretical frame

Applying the Health Services Utilization Model (HSU) [29–31], predisposing, enabling, and need components were selected as predictors of never having a mammogram. To address neglect of acculturation in the HSU, acculturation components were added to the model. Many prior studies using the HSU were also aware of this and tried to fuse acculturation as predisposing components [6, 7, 32]. Therefore, the current study treated acculturation as predisposing components. The HSU treated ethnicity as a predisposing demographic characteristic, but solely as a main effect (i.e., hypothesizing ethnic differences in health service utilization) [31]. Thus, a moderating role of ethnicity was added to the theoretical model to achieve one of the purposes of the current study. In order to understand the moderator role of ethnicity, predisposing acculturation and enabling components were selected for possible moderation of ethnicity based on the following rationales. The three ethnic groups are likely to have diverse immigration and acculturation experiences, which could lead to variation in never having a mammogram [33]. Life cycle characteristics of the three groups are culturally and socially constructed based on their unique situations or environments [33], so the three groups could show different patterns while engaging with U.S. culture; this could lead to ethnic variability in predictors of never having a mammogram. In addition, Andersen (1995)[29] mentioned that enabling components are potentially mutable; they could provide targets for future ethnic-tailored intervention.

### Purposes of the current study

For objective 1, this study examined significant predictors of never having a mammogram among Chinese, Vietnamese, and Korean immigrant women living in California and age 40 years and older. Predictors were predisposing demographic characteristics (including ethnicity), predisposing acculturation, enabling, and need components. For objective 2, this study explored whether relationships between enabling components and acculturation components and odds of never having a mammogram vary across Chinese, Vietnamese, and Korean immigrant women.

## Materials and methods

### Data

Using California Health Interview Survey (CHIS) data, Chinese, Vietnamese, and Korean immigrant (i.e., foreign-born) women age 40 years or older who lived in California at the time of data collection were selected. Merged (2005-2007-2009-2011) CHIS data were utilized to have a larger sample for each of the three ethnic groups. The CHIS is a population-based random digit dialing survey of California residents age 18 or older (conducted biennially until 2012) [34]. The four waves were chosen because each wave (a) contained measures needed to test the HSU model and (b) preceded the March 2010 passage of the Patient Protection and Affordable Care Act (ACA) or its widespread implementation. Chi-square analysis of never having a mammogram by survey year confirmed no significant differences across these four waves, allowing them to be merged. Because of the complex sampling design of CHIS, replicate weights were applied for analyses. “Replicate weights allow a single sample to simulate multiple samples, thus generating more informed standard error estimates that mimic the theoretical basis of standard errors while retaining all information about the complex sample design” (p. 6) [35]. These standard errors provide more precise confidence intervals and significance tests [35]. Final sample size was N = 3,710 (weighted N = 1,710,233), consisting of 1,389 Chinese (weighted n = 944,000), 1,094 Korean (weighted n = 358,000), and 1,227 Vietnamese (weighted n = 405,000). Case Western Reserve University IRB determined this study (STUDY 20180176) qualified as “not human subjects research,” under the New Revised Common Rule covering secondary analysis of publicly-available de-identified data.

#### Rationale for selecting the three ethnic groups

Chinese, Vietnamese, and Korean women were selected because they are among the top five immigrant groups in the U.S. [36]. These groups also are all East Asians, in terms of geographic proximity and historical contact. The current study tried to reduce ethnic heterogeneity in order to conserve statistical power, yet still include groups where breast cancer screening disparities could be anticipated based on prior research. The current study also selected only immigrant women because Asian immigrants face a number of barriers to health care access and utilization [37], and there are documented disparities in rates of lifetime or recent mammography [5], breast cancer stage at diagnosis, and survival rate [4] between foreign-born and US-born Asian women.

#### Rationale for targeting women age 40 years or older

There is a lack of consensus about the age at which to begin mammography screening for women with average risk. ACS (2017) recently issued new guidelines and it recommended that women with an average risk of breast cancer should start having mammograms at age 45 and continue once a year until age 54, then every other year for as long as they are healthy and likely to live another 10 years [38]. The United States Preventive Services Task Force (USPSTF, 2012) recommends that women aged 50–75 years should be screened for breast cancer by mammography every 2 years [39]. However, the guidelines from ACS (2017) and USPSTF (2012) seem unlikely to be accepted by other cancer organizations since physicians have recommended mammograms every year, starting at age 40: for example, the National Comprehensive Cancer Network (NCCN), an alliance of prominent cancer centers, has recommended mammograms every year starting at age 40. The American College of Obstetricians and Gynecologists (ACOG) has also recommended mammograms every year or two from age 40 to 49 and every year after that [40]. It seems that experts are still debating the balance between the benefits of this test against its potential harms for women. In addition, physicians suggest that there is no specific age at which mammography screening should be discontinued: the decision to stop regular mammography must be individualized with regard to the potential benefits and harms of mammography within the context of overall health status [41]. Even though professionals are still debating the best timing and intervals of mammography screening, there is little debate about benefits of having a mammogram as breast cancer preventive care. Following the guidelines from the NCCN [42] and ACOG [40], the current study targeted Asian immigrant women 40 years or older.

Also, considering the importance of mammography as preventive behavior, the current study focused on never having a mammogram (rather than recent mammogram). Individuals who have never had a mammogram during their lifetime could be more exposed to risks of breast cancer diagnosis at an advanced stage. Further, predictors of never having a mammogram have been understudied compared to predictors of recent mammography.

### Measures

The outcome was never having a mammogram, based on responses to “Have you ever had a mammogram?” (yes = 0, no = 1). Details about the predictors—predisposing socio-demographic characteristics and acculturation, enabling, and need components—are presented in Table 1. The measure of U.S. citizenship included only non-citizen and naturalized citizen because all respondents were foreign-born. In order to control for potential differences across the merged waves, survey year was included as a predictor.

**Table 1:** Measures.

	Measures	Coding
**Predisposing socio-demographics**	Ethnicity	Dummy 1: Chinese; Dummy 2: Korean; Vietnamese (Ref)
	Age	Dummy 1: 40–49 years; Dummy 2: 50–59 years; Dummy 3: 60–69 years; 70–85 years (Ref)
	Marital status	Widowed, divorced, separated, or never married = 1; Married or living with partner = 0 (Ref)
	Education^a^	0 = no formal education; 1 = Grade 1–8; 2 = Grade 9–11; 3 = Grade 12/H.S diploma; 4 = Some college; 5 = Vocational school; 6 = AA or AS degree; 7 = BA or BS degree; 8 = Some graduate school; 9 = MA or MS degree; 10 = PhD or equivalent.
	Federal Poverty Level	Dummy 1: 0–99% of FPL; Dummy 2: 100–199% of FPL; Dummy 3: 200–299% of FPL; 300% of FPL (Ref)
	Employment	Currently not working = 1; currently working = 0 (Ref)
	Family structure	Single or married with no children = 1; Single or married with children = 0 (Ref)
**Predisposing acculturation**	English proficiency	Speak English not well or not at all = 1; Very well or well = 0 (Ref)
	U.S. Citizenship	Non-citizen = 1; Naturalized citizen = 0 (Ref)
	Years lived the U.S.	Dummy 1: less than 10 years; Dummy 2: 10–14 years; 15 years and longer (Ref)
**Enabling**	Insurance type	Dummy 1: uninsured; Dummy2: public insurance; private or employment based (Ref)
	Location	Non urban = 1; Urban = 0 (Ref)
	Number of physician visits in past 12 months^b^	0 to 10+
	Communication problem with physicians	Have problem = 1; No problem = 0 (Ref)
**Need**	Perceived general health status ^a^	poor = 0, fair = 1, good = 2, very good = 3, excellent = 4;
	Number of chronic illnesses^c^	0 to 5 (Asthma [yes = 1, no = 0], diabetes [yes = 1, no = 0], High Blood Pressure [yes = 1, no = 0], Heart Disease [yes = 1, no = 0], Heart failure or congestive heart failure [yes = 1, no = 0])
	Psychological distress (Kessler’s Psychological Distress Scale)^d^	**During the past 30 days:** About how often did you feel nervous?; About how often did you feel hopeless?; About how often did you feel restless or fidgety?; How often did you feel so depressed that nothing could cheer you up?; About how often did you feel that everything was an effort?; About how often did you feel worthless? [0 = None, 1 = A little, 2 = Some, 3 = Most, 4 = All] 0 (low) to 24 (high)

Ref = reference group.

^a^ Mean-centered for logistic regression analyses.

^b^ Natural log transformed (-0.69 to 2.35) and mean centered for logistic regression analyses.

^c^ Natural log transformed (-.69 to 1.70) and mean centered for logistic regression analyses.

^d^ Natural log transformed (-0.69 to 3.19) and mean centered for logistic regression analyses. Cronbach alpha = 0.85.

### Analysis strategies

STATA 14.0 [43] was used for analyses. Univariate frequencies, descriptive statistics, histograms, and bivariate scatterplots were examined for outliers, adequate variability, and skewness and kurtosis. Assumptions and conditions for logistic regression were evaluated: non-collinearity (Pearson r < 0.80 or Variance Inflation Factor [VIF] < 10), lack of sparseness (i.e., cell counts > 0 and < 20% of cells with expected frequencies < 5 in cross-tabulations between predictors), non-separation of data (cell counts > 0 in cross-tabulations between predictors and the outcome), and absence of influential multivariate outliers (Cook’s distance value < 1). Errors were assumed independent because the CHIS sampling design is cross-sectional and non-clustered (i.e., independent respondents in each wave). Before examining the objectives of the current study, bivariate analyses (Chi-square or ANOVA) were conducted to investigate differences on the outcome and the predictors by ethnicity.

For objective 1, multivariate logistic regression including main effects was performed, and for objective 2, multivariate logistic regression including both main effects and interaction terms was conducted. Since replicate weighted survey data in STATA do not allow hierarchical entry of predictors, this study tested six logistic regression models: Model 1: socio-demographic characteristics; Model 2: model 1 components + acculturation measures; Model 3: model 2 components + enabling components; Model 4: model 3 + need components; Model 5: model 4 components + CHIS survey year; Model 6: model 5 components + interactions between ethnicity and acculturation, and between ethnicity and enabling components. To evaluate block changes of adding each component, an adjusted F statistic was applied [43, 44]. For goodness-of-fit of each model, the Omnibus test value is reported. For significance of predictors, the odds ratio (OR) was considered with its 95% confidence interval.

When testing interaction terms involving a continuous predictor (Model 6), interaction effects were created using an alternative method described by Cohen et al. (2003, pp. 380–382) that estimates interaction terms for all three ethnicities, instead of the traditional exclusion of the reference group [45]. Based on Model 6 initial findings, non-significant interaction terms were trimmed and the logistic regression re-estimated (Trimmed Model 6). Because of the alternative method of estimating interaction terms, changes in model fit cannot be compared between the final model with interactions and prior models without interactions.

## Results

Preliminary screening showed adequate variability on the predictors and outcome. No influential univariate or multivariate outliers were observed (maximum Cook’s distance = 0.72). Among the continuous predictors, number of physician visits, number of chronic illnesses, and psychological distress were skewed. Thus, natural log transformation was applied to these three variables. No problem was detected with separation or multicollinearity (maximum r = |.66|, maximum VIF = 4.53). Only one problem was detected with sparseness, in a cross-tabulation between ‘communication problem with physician’ and ‘ethnicity’; consequently, this interaction effect was omitted from Model 6.

### Bivariate differences by ethnicity

Table 2 shows estimated differences by ethnicity (based on weighted N) for the outcome and predictors: most of the predisposing socio-demographic characteristics, predisposing acculturation, enabling, and need components were significantly associated with ethnicity. A total of 187,000 women (weighted) in this sample (10.9%) were estimated to have never had a mammogram. Korean women had significantly higher estimated prevalence of never having a mammogram than either Chinese or Vietnamese women.

**Table 2:** Outcome and predictors by ethnicity (unweighted N = 3,710; weighted N = 1,710,233).

	Chinese			Korean			Vietnamese			X^2^	F	P-value
	Weighted		Unweighted	Weighted		Unweighted	Weighted		Unweighted			
**Lifetime Mammography (n, %)**												
Never had	94,000	10.0%	118	58,000	16.1%	183	35,000	8.6%	79	7.96		**0.023**
Ever had	850,000	90.0%	1,271	300,000	83.9%	1,044	370,000	91.4%	1,015			
**Age (n, %)**												
40–49	350,000	36.9%	431	130,000	34.6%	314	140,000	34.3%	306	42.71		**0.001**
50–59	250,000	26.9%	402	81,000	22.5%	221	130,000	32.2%	335			
60–69	140,000	15.3%	264	81,000	22.5%	305	96,000	23.7%	275			
70–85	200,000	20.9%	292	74,000	20.4%	387	40,000	9.9%	178			
**Family Structure (n, %)**												
No children	650,000	68.8%	968	270,000	73.9%	944	250,000	62.6%	733	7.45		**0.029**
Have children	290,000	31.2%	421	94,000	26.1%	283	150,000	37.4%	361			
**Marital Status (n, %)**												
Unmarried	230,000	24.0%	410	110,000	29.1%	470	120,000	28.5%	388	3.99		0.146
Married	720,000	76.0%	979	260,000	70.9%	757	290,000	71.5%	706			
**Education (M, SE)**	4.82	0.11		4.73	0.12		3.19	0.13			164.99	**< .001**
95% CI	4.61, 5.04			4.48, 4.97			2.93, 3.46					
**FPL**^a^**(n, %)**												
0–99%	160,000	17.2%	259	84,000	23.2%	358	150,000	36.4%	439	81.28		**< .001**
100–199%	210,000	22.8%	297	68,000	18.8%	250	120,000	30.7%	315			
200–299%	130,000	13.3%	174	52,000	14.3%	167	36,000	8.9%	111			
300% above	440,000	46.7%	659	160,000	43.7%	452	98,000	24.0%	229			
**Employment (n, %)**												
Unemployed	500,000	52.9%	731	220,000	61.1%	836	250,000	60.5%	720	8.65		**0.017**
Employed	440,000	47.1%	658	140,000	38.9%	391	160,000	39.5%	374			
**English Proficiency (n, %)**												
Not well/ not at all	510,000	54.5%	725	270,000	74.4%	915	300,000	74.0%	810	55.22		**< .001**
Well/very well	430,000	45.6%	664	92,000	25.6%	312	110,000	26.1%	284			
**U.S. Citizenship (n, %)**												
Non-citizen	150,000	15.8%	195	120,000	32.0%	295	76,000	18.7%	155	30.03		**< .001**
Naturalized	790,000	84.2%	1194	250,000	68.0%	932	330,000	81.3%	939			
**Years Lived In the U.S. (n, %)**												
< 10 yrs	120,000	12.8%	147	52,000	14.3%	138	54,000	13.2%	116	5.72		0.250
10–14 yrs	110,000	12.0%	169	32,000	8.8%	105	54,000	13.2%	144			
15 yrs+	710,000	75.1%	1073	280,000	77.0%	984	300,000	73.6%	834			
**Insurance Type (n, %)**												
Uninsured	69,000	7.3%	123	110,000	30.1%	249	57,000	13.9%	131	57.32		**< .001**
Public insurance	330,000	35.5%	489	120,000	34.5%	599	180,000	43.3%	595			
Other	540,000	57.1%	777	130,000	35.5%	379	170,000	42.7%	368			
**Location (n, %)**												
Non-urban	250,000	26.6%	372	77,000	21.4%	269	63,000	15.5%	113	13.05		**0.003**
Urban	690,000	73.4%	1017	280,000	78.6%	958	340,000	84.5%	981			
**MD Visits**^**b**^^,^ ^**c**^ **(M, SE)**	2.97	0.08		3.54	0.16		3.40	0.16			21.86	**< .001**
95% CI	2.80, 3.14			3.23, 3.86			3.07, 3.72					
**Communication Problem with MD** ^**b**^ **(n, %)**												
Have problem	79,000	8.4%	118	27,000	7.5%	82	64,000	15.7%	134	11.97		**0.004**
No problem	860,000	91.6%	1,271	330,000	92.5%	1,145	340,000	84.3%	960			
**Perceived Health (M, SE)**	1.98	0.04		1.67	0.07		1.35	0.07			126.1	**< .001**
95% CI	1.89, 2.07			1.53, 1.80			1.21, 1.49					
**# of Chronic Illnesses**^**c**^ **(M, SE)**	0.53	0.03		0.55	0.04		0.58	0.05			18.00	**< .001**
95% CI	0.47, 0.59			0.48, 0.62			0.49, 0.68					
**Psychological Distress**^**c**^ **(M, SE)**	2.97	0.15		5.06	0.26		3.06	0.34			21.86	**< .001**
95% CI	2.67, 3.26			4.53, 5.58			2.92, 4.29					
**Survey year**												
2005	220,000	23.6%	345	87,000	23.9%	217	110,000	26.9%	156	9.75		0.183
2007	220,000	23.4%	375	84,000	23.3%	257	82,000	20.0%	132			
2009	240,000	25.9%	305	66,000	18.3%	410	94,000	23.0%	457			
2011	250,000	27.0%	364	120,000	34.5%	343	120,000	30.0%	349			

Weighted N and % are presented. Unweighted N is presented as well. Except for continuous measures, Chi-square, F statistic, and p-value were generated by replicate weighted data analyses. Weighted data generates SE with mean instead of SD. P-values from ANOVA for continuous measures were generated from unweighted data. Education could range from no formal education = 0 to Ph.D. or equivalent = 10.

95% CI = 95% confidence interval. Significant differences are in bold-type.

^a^ FPL = Federal Poverty Level

^b^ MD = Physician

^c^ Untransformed in Table 2

Other notable ethnic differences were that Chinese women had a higher percentage who said they were employed, who said they speak English well or very well, and they reported fewer mean physician visits. Korean women had a higher percentage who were childless, not U.S. citizens, with no health insurance, and reported the highest mean psychological distress. Vietnamese women tended to be younger, had the lowest education level, were poorer, had a higher percentage in urban areas, a higher percentage reporting a problem communicating with physicians, poorest perceived health on average, and higher mean number of chronic diseases. The three groups did not differ on marital status or years lived in the U.S.

### Multivariate analyses

Table 3 shows logistic regression findings for objective 1 (testing main effects of predictors). Across models, at least one predisposing socio-demographic characteristic, predisposing acculturation measure, and enabling component significantly predicted the odds of never having a mammogram. However, need components were never significant predictors. Once all other predictors were controlled in the final main effects model (Model 5), no statistically significant ethnic differences in odds of never having a mammogram were found. Compared to the oldest women (70–85 years), middle-aged (50–59 years) and young-old women (60–69 years) had lower odds of never having a mammogram. However, women whose age was 40–49 years were not significantly different from the oldest women (70–85 years). In other words, women ages 40–49 or 70–85 years were more vulnerable to never having a mammogram. Unmarried (compared to married) women and non-U.S. citizens (compared to naturalized citizens) had higher odds of never having a mammogram. Among enabling components, the odds of never having a mammogram were lower as the number of doctor visits got higher.

**Table 3:** Multivariate logistic regression models predicting odds of never having a mammogram for the three groups of Asian immigrant women (unweighted N = 3,710; weighted N = 1,710,233).

	MODEL 1			MODEL 2			MODEL 3			MODEL 4			MODEL 5		
	OR	95% CI		OR	95% CI		OR	95% CI		OR	95% CI		OR	95% CI	
Constant	0.14	0.04	0.48	0.12	0.04	0.41	0.09	0.03	0.27	0.09	0.30	0.30	0.12	0.04	0.38
**Predisposing Socio-Demographics**															
Chinese (Ref = Vietnamese)	1.16	0.70	1.93	1.15	0.67	1.95	1.12	0.66	1.90	1.17	0.69	1.99	1.21	0.72	2.04
Korean (Ref = Vietnamese)	**2.06**	**1.23**	**3.46**	1.73	0.99	3.03	1.67	0.93	3.00	**1.83**	**1.02**	**3.30**	1.80	1.00	3.22
40–49 yrs (Ref = 70–85 yrs)	1.32	0.67	2.60	1.12	0.59	2.13	1.07	0.49	2.31	1.00	0.42	2.43	1.01	0.42	2.43
50–59 yrs (Ref = 70–85 yrs)	**0.42**	**0.21**	**0.85**	**0.39**	**0.19**	**0.80**	**0.38**	**0.17**	**0.85**	**0.37**	**0.15**	**0.90**	**0.37**	**0.15**	**0.89**
60–69 yrs (Ref = 70–85 yrs)	**0.36**	**0.19**	**0.68**	**0.37**	**0.20**	**0.70**	**0.38**	**0.19**	**0.78**	**0.37**	**0.18**	**0.78**	**0.36**	**0.17**	**0.75**
No children (Ref = Have children)	0.65	0.38	1.11	0.69	0.41	1.18	0.69	0.40	1.19	0.69	0.40	1.20	0.68	0.39	1.19
Not married (Ref = Married)	**1.77**	**1.11**	**2.83**	**1.78**	**1.12**	**2.82**	**1.82**	**1.13**	**2.92**	**1.80**	**1.11**	**2.92**	**1.74**	**1.08**	**2.82**
Education (Mean centered)	0.96	0.88	1.05	0.97	0.88	1.07	0.98	0.89	1.07	0.99	0.90	1.08	0.98	0.90	1.08
0–99% of FPL (Ref = 300+%)	0.75	0.38	1.49	0.75	0.39	1.45	0.79	0.43	1.45	0.76	0.41	1.42	0.75	0.40	1.41
100–199% of FPL (Ref = 300+ %)	0.92	0.51	1.65	0.91	0.49	1.70	0.91	0.48	1.70	0.88	0.46	1.69	0.93	0.48	1.80
200–299% of FPL (Ref = 300+ %)	0.80	0.41	1.57	0.81	0.42	1.56	0.80	0.42	1.54	0.77	0.40	1.50	0.79	0.41	1.50
Unemployed (Ref = Employed)	1.47	0.92	2.33	1.34	0.84	2.15	1.30	0.81	2.10	1.32	0.83	2.08	1.32	0.85	2.06
**Predisposing Acculturation**															
Poor at English (Ref = well/very well)				0.95	0.59	1.51	0.88	0.56	1.38	0.83	0.52	1.33	0.82	0.51	1.33
Non-citizen (Ref = Naturalized U.S. Citizen)				**2.62**	**1.48**	**4.64**	**2.52**	**1.43**	**4.43**	**2.53**	**1.42**	**4.51**	**2.56**	**1.44**	**4.55**
Lived <10 yrs in U.S. (Ref = 15 years+)				0.77	0.42	1.40	0.73	0.41	1.31	0.71	0.40	1.28	0.72	0.40	1.29
Lived 10–14 yrs in U.S. (Ref = 15 years+)				1.25	0.70	2.22	1.28	0.72	2.27	1.26	0.72	2.22	1.23	0.71	2.14
**Enabling**															
Uninsured (Ref = Private insurance)							1.49	0.87	2.56	1.49	0.88	2.52	1.52	0.89	2.61
Public insurance (Ref = Private insurance)							1.19	0.63	2.28	1.21	0.62	2.36	1.22	0.64	2.33
Non-urban (Ref = Urban)							1.26	0.74	2.14	1.26	0.73	2.16	1.30	0.76	2.24
# of physician visits ^a^							**0.76**	**0.60**	**0.97**	**0.76**	**0.60**	**0.96**	**0.75**	**0.60**	**0.95**
Communication problem with physician (yes)							1.11	0.54	2.30	1.12	0.54	2.35	1.13	0.54	2.34
**Need**															
Perceived health ^b^										0.89	0.71	1.12	0.89	0.71	1.11
# of chronic illnesses ^a^										0.81	0.53	1.26	0.82	0.54	1.26
Psychological distress ^a^										0.89	0.73	1.08	0.89	0.73	1.08
**Survey Years (**Ref = CHIS 2011)															
2005													0.68	0.43	1.07
2007													0.71	0.40	1.25
2009													0.57	0.30	1.07
Block Test	F (12) = 6.91, p< .001			F (4) = 3.57, p = .010			F (5) = 1.78, p = .027			F (3) = 1.02, p = .390			F (3) = 1.41, p = .247		
Omnibus Test	F = 6.91, p< .001			F = 7.89, p< .001			F = 7.64, p< .001			F = 6.58, p< .001			F = 6.41, p< .001		

Ref = Reference Group. Odds Ratio (OR) whose Confidence Interval (CI) does not include 1.00 is statistically significant at p < .05. Significant Odds Ratio and its 95% CI is in bold-type. Estimates are based on weighted N. Each model was tested separately, because STATA does not allow hierarchical entry of predictors with replicated weighted survey data.

^a^ Natural log transformed and mean centered for multivariate logistic regression analyses.

^b^ Mean centered

For objective 2, a total of 6 interactions were initially tested (ethnicity by three acculturation measures and ethnicity by three enabling measures). Table 4 presents the trimmed Model 6 including the sole significant interaction effect (number of physician visits by Chinese ethnicity) and main effects. For Chinese women, for every one unit higher on logged physician visits, the odds of never having a mammogram were 0.67 times as high compared to Chinese women one unit lower. This means that the odds of never having a mammogram significantly decreased with additional physician visits for Chinese women. For Korean and Vietnamese women, there was no significant interaction with number of physician visits, though.

**Table 4:** Final trimmed multivariate logistic regression with significant interaction effects by ethnicity (unweighted N = 3,710; weighted N = 1,710,233).

	OR	95% CI	
**Constant**	0.13	0.04	0.38
**Main Effects for Socio-demographic**			
Chinese (Ref = Vietnamese)	1.11	0.66	1.87
Korean (Ref = Vietnamese)	1.77	1.00	3.12
40–49 yrs (Ref = 70–85)	1.02	0.43	2.41
50–59 yrs (Ref = 70–85)	**0.38**	**0.16**	**0.90**
60–69 yrs (Ref = 70–85)	**0.36**	**0.17**	**0.76**
No children (Ref = have children)	0.69	0.39	1.21
Not married (Ref = Married)	**1.73**	**1.07**	**2.81**
Education ^a^	0.99	0.90	1.08
0–99% of Federal Poverty Level (Ref = 300%+ of FPL)	0.73	0.39	1.37
100–199% of Federal Poverty Level (Ref = 300%+ of FPL)	0.92	0.49	1.75
200–299% of Federal Poverty Level (Ref = 300%+ of FPL)	0.77	0.41	1.47
Unemployed (Ref = Employed)	1.30	0.84	2.03
**Main Effects for Acculturation**			
Poor at English (Ref = Well/Very well)	0.81	0.50	1.33
**Non- citizen (Ref = naturalized citizen)**	**2.56**	**1.44**	**4.56**
Lived < 10 years in the U.S. (Ref = longer than 15 years)	0.74	0.42	1.31
Lived 10–14 years in the U.S. (Ref = longer than 15 years)	1.24	0.71	2.17
**Main Effects for Enabling Components**			
Uninsured (Ref = employment based or private)	1.58	0.92	2.72
Public insurance (Ref = employment based or private)	1.24	0.66	2.35
Non-urban (Ref = urban)	1.32	0.76	2.28
Communication problem with physician (Ref = No problem)	1.15	0.56	2.35
**Main Effects for Need**			
Perceived health ^a^	0.89	0.71	1.11
Number of chronic illnesses^b^	0.81	0.53	1.25
Psychological distress^b^	0.89	0.73	1.08
**Survey year (Ref = 2011)**			
2005	0.68	0.43	1.07
2007	0.70	0.39	1.23
2009	0.57	0.31	1.07
**Interaction Effects for Ethnicity by Number of Physician Visits** ^**b**^^,^ ^**c**^			
Chinese × physician visits	**0.67**	**0.46**	**0.98**
Korean × physician visits	0.86	0.63	1.19
Vietnamese × physician visits	0.84	0.60	1.16
**Omnibus Test**	F = 6.19, p< .001		

Outcome is never had a mammogram (coded 1)

Ref = Reference group.

This final model presents the estimates after removing non-significant interaction effects and re-estimating the model. Odds Ratio (OR) whose Confidence Interval (CI) does not include 1.00 is statistically significant at p < .05 is in bold-type.

^a^Mean centered

^b^ Natural log transformed and mean centered

^c^ Standardized guideline [45] was used for testing the moderating effect of ethnicity on the continuous measure: “the simple main effect of the continuous variable involved in an interaction term should be excluded, and the simple main effect for the reference group should be represented in the intercept” (p. 382).

With the interaction effect in the model, all other main effects remained the same as before: significant differences in odds of never having a mammogram by age, marital status, and citizenship status, and no significant effects for need factors. In the final model, protective factors were more physician visits, but only for Chinese immigrant women, and middle and young-old ages. In contrast, being unmarried and not a U.S. citizen were risk factors.

## Discussion

Korean women had the highest percentage never having a mammogram in bivariate analysis and higher odds than Vietnamese women of never having a mammogram in some multivariate analyses. The bivariate results are consistent with research by Ma et al. (2009, 2012), although the percentages vary. According to Ma et al. (2009), among Asian subgroups, Chinese women reported the lowest percentage and Korean women the highest percentage of never having a mammogram (20.10% of Chinese, 28.36% of Vietnamese, and 30.23% of Koreans) [24]. Similarly, in Ma et al. (2012) Korean women again had the highest percentage of never having a mammogram (28.78%, compared to 26.90% for Vietnamese and 19.32% for Chinese) [15]. In the current study, an estimated 10.0% of Chinese, 8.6% of Vietnamese, and 16.1% of Korean immigrant women never had a mammogram. Even though the percentages of never having a mammogram are different between Ma et al.’s (2009) and (2012) studies and the current study, Korean women had the highest percentage of never having a mammogram in all three studies. The differences in percentages could be caused by several method-related factors (e.g., different geographic areas in data collection, total sample and sub-population sample sizes, analytic strategy).

Possibly, Korean immigrants are reluctant to use healthcare services in the U.S. because they are concerned about the lack of physicians’ cultural understanding of Koreans [46]. Pew Research Center (2013) [47] mentioned that “Koreans have the highest self-employment rate among U.S. Asians” (p.51). Self-employed immigrants might have less chance to visit clinics for breast cancer screening due to their tight schedule for managing their own business and lack of health insurance. The present study also showed a higher percentage of Korean women reported being uninsured than either other group, but the measure of employment status in the present study did not distinguish self-employment from being employed by others.

The mammography disparity for Korean women was significant in some multivariate analyses but not others, depending on what other predictors were controlled. The difference between Korean and Vietnamese women was not significant, for example, when acculturation and enabling components were first entered in the model, perhaps due to the fact that, in bivariate analyses, several differences were evident between Korean and Vietnamese women on these components. After need was controlled, however, Korean women again showed significantly higher odds of never having a mammogram. These fluctuating findings are consistent with the view that ‘ethnicity’ should be understood as representing a complex network of factors that influence Asian immigrant women’s lifetime mammography behavior.

The current study and prior studies suggest that findings regarding Asian ethnic disparities in never having a mammogram may depend not only on what predisposing socio-demographic or acculturation, enabling, and need components are considered, but also on how these components are operationalized.

Lee et al. (2014) [48] examined predictors of recent breast cancer screening of Chinese, Vietnamese, and Korean (reference group) immigrant women and found no significant difference in mammography behavior between Vietnamese and Korean women when controlling for acculturation (multidimensional Suinn-Lew Asian Self Identity Acculturation Scale) and socio-demographic characteristics (age, insurance status, marital status, income). Kandula et al. (2006) examined key predictors of recent mammography of diverse racial/ethnic groups (Non-Hispanic white [reference group], Chinese, Filipino, Vietnamese, Japanese, Korean, South Asian, or Cambodian) and found that Chinese, Korean, and Filipino had significantly lower odds of having a mammogram controlling for socio-demographic characteristics and enabling components (e.g., insurance, having a usual source of care). However, adjusting for acculturation components (nativity, years in the United States, and English language) attenuated the relation between Asian ethnicity and lower odds of having a mammogram [49]. Kandula et al.’s (2006) study explains that Asian subgroups’ mammography behavior could be strongly influenced by complex or different acculturation status of those groups. In addition, these prior studies suggest that findings regarding disparities may also reflect the choice of reference group.

The lower prevalence of never having a mammogram in the 50–59 and 60–69 years could be associated with the breast cancer incidence rate in ages 50 years and older in the U.S. According to ACS (2017), breast cancer incidence rates have increased among American women over the age of 50 during the most recent period (2005–2014) [2]. In Asian/Pacific Islander women, the incidence rate rises steeply after about age 45 and peaks about age 65 [2].

Unmarried middle-aged and older women have been identified in prior research as one of the underserved populations showing higher odds of never having a mammogram [50, 51] than their counterparts. Similarly, the present study found unmarried women had 73% higher odds of never having a mammogram. Marriage may confer health security because it can create opportunities for health insurance through a spouse [52]. Additionally, supportive relationships from marriage have shown positive effects on health [53, 54]. Tangible support from marriage (e.g., reminding the spouse to undergo screening or driving the spouse to hospitals/clinics) could be responsible for the healthcare advantage of married women [55].

This study found that noncitizens had two and a half times higher odds of never having a mammogram than naturalized U.S.-citizens, which is consistent with Ryu et al.’s (2013) finding [14]: even after controlling for poverty level, education, English proficiency, and years lived in the U.S., there was a significant effect of non-citizenship on never having a mammogram. It is important to uncover the underlying factors that can cause vulnerability of noncitizens to never having a mammogram [56]. Limited access to healthcare services of noncitizens is one possibility [57, 58]. Many immigrants without U.S. citizenship also lack knowledge about available resources (e.g., free screenings, Medicare, Medicaid, and programs from insurance companies) [56].

Having fewer physician visits was a significant predictor of never having a mammogram in all the main effects models. Prior studies found that Asian women having a usual source of healthcare [16] and more chances to check health status regularly [20, 32] were less vulnerable to never having a mammogram. People having limited healthcare access could have less chance to visit their primary physicians [59, 60], which in turn could limit their knowledge of the importance of mammography.

Notably, after interaction by ethnicity was tested, number of physician visits had a significant effect on never having a mammogram among Chinese women only. According to Simon et al.’s (2017) study, Chinese women in the U.S. were willing to adhere to physicians’ directions and guidelines [61]. Chinese women were reluctant to ask questions to physicians [62]; they would rather have a higher level of trust in physicians in clinical encounters [63, 64]. Le et al. (2014) said Chinese were more likely to consider eastern medicine to take care of their health compared to other Asian groups (Chinese = 52%, Korean = 27%, Vietnamese = 27%, p < .01), so they were less likely to visit clinics. However, once Chinese had a chance to be exposed to western medicine, they (93%) were more likely to believe that western medicine was effective in caring for their health, compared to Korean (76%) or Vietnamese (79%) [65].

## Study limitations and strengths

This study has several limitations. Although California is home to a large Asian population [66], generalizability of study results to other U.S. locations or nationwide is unknown. Comparisons between socio demographic characteristics of the present sample and national samples are difficult to make: there is a growing but still limited body of national data on Asians, national surveys often contain a small number of Asian respondents and frequently report aggregated results (i.e., for Asians as a whole), and they typically do not present socio demographic characteristics by nativity or gender. A few comparisons can be drawn with national studies that reported results separately for Chinese, Korean, and Vietnamese respondents. According to Pew Research Center (2013), for example, 2010 average annual household income range in the U.S. for Chinese, Korean, and Vietnamese respondents was from $50K to $65K [67]. For the current study, average annual household income levels of the three groups covered a slightly broader range: $70K, $56K, and $40K for Chinese, Korean, and Vietnamese, respectively. Considering education levels of Asian subgroups in the U.S., 51% of Chinese, 53% of Koreans, and 26% of Vietnamese were educated with a bachelor degree or higher [67], while 41% of Chinese, 41% of Koreans, and 17% of Vietnamese immigrant women in the current study were educated with a bachelor degree or higher.

A study [68] using the National Health Interview Survey (1992–1995) to examine self-rated health reported 88.6% of Chinese immigrants, 83% of Korean immigrants, and 75.8% of Vietnamese immigrants said that their health was good, very good, or excellent, while only 64% of Chinese, 55% of Korean, and 36% of Vietnamese immigrant women in the current study said their health status was good, very good, or excellent. Consequently, the sample in the current study appears to have a broader range of average household income, lower attainment of a bachelor degree or higher, and worse self-rated health than national statistics. The extent to which these differences reflect gender, nativity, or geographic location is not known. Thus, future studies need to consider broader geographic representation and comparison between foreign-born and U.S.-born women to test generalizability of the present findings.

In addition, need components were not significant predictors in the present study, which is counterintuitive to the HSU model but consistent with prior studies’ findings [9, 11, 22, 69]. Some of the need factors used in this study and prior studies (e.g., perceived general health status or number of chronic illnesses) might be too general to predict never having a mammogram. Although one prior study examined effects of cancer-specific need indicators on both lifetime mammography and recent mammography [13], family cancer history significantly predicted recent mammography only. Also, different time frames between need components and the outcome might explain the nonsignificant effects of the need components in this study. For example, psychological distress asked about ‘past 30 days,’ while never having a mammogram asked about their lifetime behavior.

Despite limitations, this study offers valuable contributions to research on healthcare for female Chinese, Korean, and Vietnamese immigrants. First, unlike research that analyzes Asian women in the aggregate, this study provided important information related to the prevalence of never having a mammogram of three groups of Asian immigrants. In all three groups, a substantial minority (ranging from 8.6% of the Vietnamese women to 16.0% of the Korean women) was estimated to have never had a mammogram. Second, the study provides baseline results obtained prior to enactment or wide implementation of the ACA. Building on the current study, future studies could examine whether the ACA facilitates lifetime mammography of Asian immigrant women or not. Last, by modifying the HSU model to integrate both general factors, which can be used to explain health care service use in any population, and specific factors germane to the immigrant group (the acculturation component), the present study documented both general disparities for never having a mammogram (higher odds for women who were not married and who were ages 40–49 or 70 and older) and disparity specific to immigrant status (higher odds for non-citizens).

## Future research suggestions

The current study provides several suggestions for future research. First, the significant interaction between number of physician visits and being Chinese provides some rationale for continuing to explore possible moderating effects of ethnicity. Second, although prior research offers insights into potential differences across Asian subgroups in their patterns of physician visits [65], there are still many things to be learned including levels of trust in physicians [63, 64], adherence to physician’s guidelines [61], or satisfaction with healthcare services in the U.S. [65]. We do not know, for example, why Vietnamese and Korean women appear to derive less benefit from physician visits when it comes to mammography. Future research also could expand investigation of moderation to investigate whether effects of predisposing sociodemographic characteristics vary by ethnicity. Last, with respect to the fluctuating disparity for never having a mammogram of Korean immigrant women (e.g., in multivariate analyses, Koreans were sometimes significantly more vulnerable to never having a mammogram and sometimes not, compared to Vietnamese), both qualitative and quantitative research on reasons for never having a mammogram among Korean and Vietnamese immigrant women is needed, and this research could be expanded beyond the HSU model to also consider internal factors (e.g., health beliefs) as well as external factors.

## Conclusion and implications

As implications, the current study highlights the need for designing tailored interventions or education programs to enhance lifetime mammography of Chinese, Vietnamese, and Korean immigrant women. For example, regular information sessions or education programs could be provided in order to increase physician visits for Chinese women [70]. For all three groups, ethnically-sensitive patient-centered communication would enable patients to exchange appropriate information with their physicians, facilitate patients’ treatment adherence, and enhance the satisfaction both of patients and physicians in their relationships [71]. Effective healthcare communication between patients and healthcare providers also is associated with enhanced preventive care delivery [72].
